# Professional integration of immigrant medical professionals through alternative career pathways: an Internet scan to synthesize the current landscape

**DOI:** 10.1186/s12960-021-00599-8

**Published:** 2021-04-17

**Authors:** Tanvir C. Turin, Nashit Chowdhury, Mark Ekpekurede, Deidre Lake, Mohammad Ali Ashraf Lasker, Mary O’Brien, Suzanne Goopy

**Affiliations:** 1 Department of Family Medicine, Cumming School of Medicine, University of Calgary, G012F, Health Sciences Centre, 3330 Hospital Drive NW, Calgary, AB T2N 4N1 Canada; 2 Department of Community Health Sciences, Cumming School of Medicine, University of Calgary, Calgary, AB Canada; 3 Alberta International Medical Graduates Association, Calgary, AB Canada; 4 School of Languages, Linguistics, Literatures and Culture, University of Calgary, Calgary, AB Canada; 5 Faculty of Nursing, University of Calgary, Calgary, AB Canada

**Keywords:** International medical graduates, Alternative career, Health and wellness, Community

## Abstract

**Background:**

There is a growing recognition that underutilization and underemployment of skilled immigrants, especially internationally trained health professionals, creates a financial burden on individuals and economic losses for the host country. Albeit a missed opportunity for both the immigrants and the receiving country, no public policy and systemic measures are in place to address this issue. Nevertheless, certain individuals and organizations have made some isolated efforts, but no synthesized knowledge is available for understanding what initiatives exist altogether and how they function. We have conducted a methodological Internet scan to identify the existing individual, private, and systemic initiatives and resources that support these health professionals. This will provide health and workforce policymakers, settlement service providers, and relevant academics with the knowledge base for potential different strategies to address this issue and guide them towards developing solution-oriented initiatives.

**Methods:**

To identify those we have systematically searched the three most popular search engines (Google, Bing, and Yahoo!) adapting the Canadian Institute for Health Information’s grey literature review protocol. We identified relevant websites per our predefined inclusion criteria, charted the data from those sources, collated, summarized, and reported the results.

**Results:**

From 280 webpages initially identified through keyword search, we included 26 in our full-page screen and extracted data from 16 finally selected webpages. We have found webpages with information on different alternative careers namely, regulated and non-regulated, available resources to pursue those careers, and what skills they have that can be transferred to the alternative careers.

**Conclusion:**

More systemic policies and IMG specific and ACP-focused employment support programmes are required. Research and development of programmes for facilitating IMGs’ alternative career support need to be increased and strengthened.

## Introduction

Underutilization and underemployment of skilled immigrants create a significant financial burden on individuals and economic losses for the host country [1, 2]. This is especially true for internationally educated health professionals, including international medical graduates (IMGs), who gained their qualifications from a medical school in a country other than the one in which they seek to professionally integrate [3, 4]. These professionals are also referred to as foreign medical graduates (FMGs), overseas trained graduates (OTGs), internationally trained physicians (ITPs), or internationally educated physicians (IEPs) [3]. They are particularly vulnerable within the host country’s labour market, as many IMGs fail to be recertified and employed in their original profession [5]. For example, in the United States, about half of the IMG candidates who applied to a residency programme were denied a position [6], and in Canada, about 80% of the IMGs who applied could not enter residency programmes in 2019 [7–9].

Against this backdrop, IMGs are often forced to look for alternative careers in order to secure their livelihood. There is no established alternative career definition for IMGs, however, or this study, we have defined alternative careers for IMGs as those non-physician health and wellness jobs where the health-related knowledge and skills of IMGs can be utilized. Some IMGs choose alternative careers so they can earn a decent living and support their family while pursuing licensure [10]. For those who decide not to pursue medical licensure and give up obtaining their licence as a practising physician, the alternative career becomes their new goal. Commencing alternative career pathways (ACPs) related to health and wellness may boost IMGs’ prospects of better integration into the labour market by increasing opportunities for them to employ their transferable skills and experience [11, 12], and thereby earn a dependable living and contribute to the host country’s economy.

There has been a reasonable amount of work undertaken regarding IMGs’ professional integration as a physician in their host countries [13–15], but the work on ACPs has been scarce. Moreover, there are many organizations that provide support and information to the IMGs (such as AIMGA, HealthForceOntario, etc.) primarily on becoming licensed physicians in Canada. But the information and resources for issues related to alternative career pathways for the IMGs in Canada, who have not been certified as physicians, is scarce [16]. Some individual efforts have been noticed on the news and other media at a personal or organizational level [17], however, no knowledge synthesis is available informing what initiatives exist altogether and how they facilitate the integration of IMGs through alternative careers. A comprehensive understanding of what alternative paths are available for the professional integration of IMGs, what information resources and capacity-building initiatives are in place, and if any, how they work is quite important. While there is scarce information in the refereed journals [16], we assumed that there might be some programmes and initiatives on a smaller scale and at individual levels that might be available on the non-academic information sources. To make an evidence-informed step ahead in this previously less explored area, we need all the information that we can avail. Therefore, we have conducted this Internet scan (I-scan) with the objective of identifying the available individual, private, and systemic initiatives and resources that support these health professionals in their pursuit of alternative careers. Having this information will allow different levels of stakeholders of this matter the preliminary knowledge to take solution-oriented research and programme initiatives.

## Methods

### Comprehensive Internet-scan

The literature on alternative career pursuit of IMGs is scarce. Further, our objective for this study is to identify existing information resources and support programmes and initiatives by individuals or organizations. However, this information is less likely to be found in academic databases and even in the 1st tier of grey literature [18, 19] such as dissertations and thesis, government and organization published reports, white papers, etc. [16]. Therefore, we have taken a systematic comprehensive Internet-scan or online-scan method to capture information from news articles, blogs, public question and answer forums, organizational websites, etc., which are sometimes referred to as 2nd and 3rd tier of the shades of grey literature [18].

#### Data sources

The target data sources for this study objectives are the websites, news articles, presentations, videos, and annual reports of different employment and immigration support-providing organizations as well as educational institution’s websites. Also, different personal websites and blogs may provide valuable information. To identify this information, we have adapted the protocol for searching grey literature by the Canadian Institute for Health Information (CIHI) [20] and the Canadian Agency for Drugs and Technologies in Health (CADTH) [21] for this study. We have initially used the National Network of Libraries of Medicine [22] suggested criteria to ensure the credibility of the data source. To ensure more rigour, we have applied the AACODS (Authority, Accuracy, Coverage, Objectivity, Date, and Significance) checklist to further evaluate and critically appraise the information obtained in the I-scan [23].

#### Search strategy

Considering the study objective and the scope of this study, and in an effort to identify maximum relevant data, we have searched web search engines (instead of indexed grey databases and individually searching organizational websites) adapting the CIHI and CADTH indicated search strategy [21, 24]. We have systematically searched the three most popular search engines, Google (Google.com, Mountain View, California, USA), Yahoo! (Yahoo.com, Sunnyvale, California, USA), and Bing (Bing.com, Microsoft Corporation, Redmond, Washington, USA), to identify relevant web-based information on professional integration of IMGs through ACPs. To avoid location and personal bias in the search engines’ results, we have used incognito mode and selected any region during the search [25]. Relevant web pages from all sources, including different organizations, sites of different news agencies, individual or organizational discussion or informational sites (i.e. blogs), online discussion boards/forums and social media platforms were included.

The following search string was used in the I-scan: (1) international medical graduates, foreign medical graduates, internationally trained health professionals; and (2) alternative career pathways. We connected each of the keywords in (1) with (2) using a Boolean operator. We also looked for further relevant information beyond the landing pages and included them if found any. Following the methodology suggested by the CIHI and CADTH, only the first 10 search engine result pages (100 results) for each search combination were included to complete a comprehensive search [21, 24].

#### Inclusion and exclusion criteria

Web pages were selected based on our predefined inclusion and exclusion criteria. First of all, we have selected only those webpages that were identified credible using the National Network of Libraries of Medicine [22] suggested criteria. We have included only those webpages that provided information on health and wellness-related professions for IMGs. No country or time restrictions were placed for the eligibility of the web pages. However, only web pages in the English language were included. Websites that provided information on healthcare professions not related to IMGs and sites with non-functioning links were excluded.

#### Web page screening and data validation

Duplicate entries were removed. The potential web pages were fully reviewed for inclusion or exclusion through a screening process. Eligibility was determined based on the information on the landing page. For example, if it is clear from the information on the landing page that the website only provides information on licensing examinations for being a practising physician in the host country, but nothing on alternative career pathways, then those were excluded. At this stage, we have used the AACODS checklist to ensure if the data is valid and relevant [23]. The screening was done by two reviewers—NC and ME.

#### Data abstraction and presentation

Information regarding web page characteristics, including web address, page title, brief description, and date searched was collected. We also abstracted the executive summary or any description provided in the websites using a predefined checklist. Search results were managed in EndNote software (Clarivate Analytics, Philadelphia, Pennsylvania, USA).

## Results

### Internet search overview

**Fig. 1:**
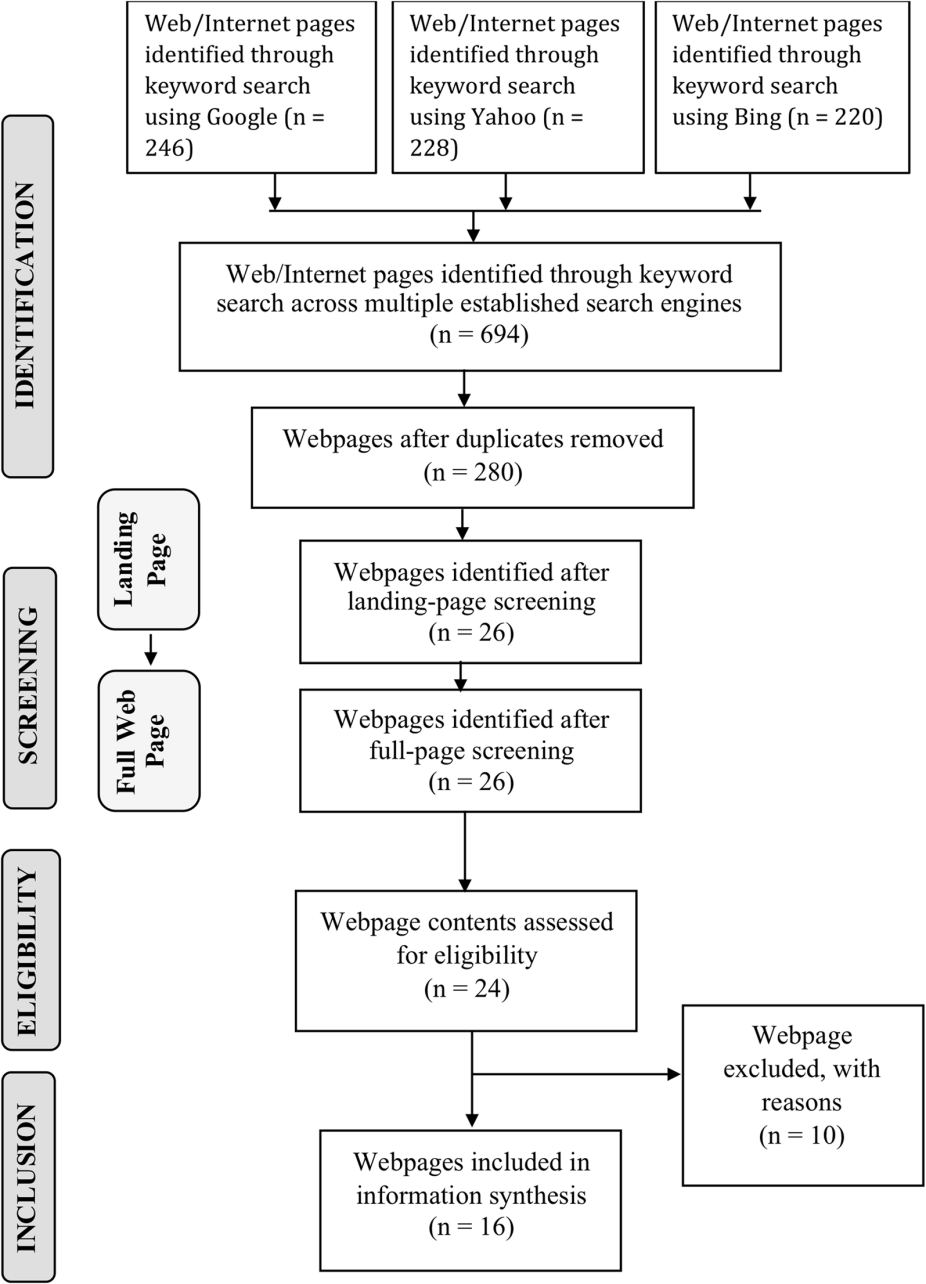
Flow diagram of the Internet search

The structured search of the three search engines (Google, Yahoo, and Bing) yielded 694 web pages. After removing duplicates, 280 web pages were selected for the landing page screening. After landing page screening, 26 web pages remained for full-page screening. Once the full screening process was completed, 16 web pages were finally selected (Fig. 1).

### Web page characteristics overview

Table 1 shows the basic charracterestics of the web-pages identified in this study. Most websites (11 of 16) were websites of organizations. All of the websites were updated within the last 3 years (2018–2020) except for one blog that was last updated in 2008. Three of the organizations were not-for-profit organizations that work and offer resources for immigrant populations, including IMGs. Two organizations exclusively assist IMGs with their professional integration. Other organizations included a human resource partner of biotech companies, a university-run bridging programme, a college offering a course for IMGs, an online institute that helps IMGs obtain research experience and publications, and one IMG-certifying organization.

**Table 1:** Website characteristics

Organization (location)	Name of web page	Web page link (date last updated)	Type of web page	Organization profile
Agincourt Community Services Association (ACSA) (Scarborough, Ontario)	Helping Newcomers Work	https://helpingnewcomerswork.ca/health-care-occupations-for-internationally-trained-newcomers/ (January 9, 2020)	Website	A non-profit, multi-service agency that talks about where to begin, elements of an employment strategy, and how one might define “success”. Their posts identify issues and opportunities, as well as “provide information and suggestions about the job search process, types of work to consider, training programmes, and upcoming opportunities.”
Alberta International Medical Graduate Association (AIMGA) (Calgary, Alberta)	Alternative Career Pathways (ACP)	https://aimga.ca/alternative-pathways-in-healthcare/ (N/R)	Website	“AIMGA is a non-profit organization dedicated to the successful integration of International Medical Graduates (IMGs).” Their vision is to “improve healthcare through the re-engagement of IMGs in Canada and support IMGs as they complete the professional requirements in order to integrate into the Canadian healthcare system.”
BioTalent Canada (Ottawa, Ontario)	Alternative Career Paths for Internationally Educated Health Professionals	https://www.biotalent.ca/alternatecareers/ (January 2020)	Website	“BioTalent Canada™ is the HR partner of and a catalyst for growth in Canada’s bio-economy. Our engagement with employers, associations, post-secondary institutions, immigrant serving agencies and service providers has built a dynamic network that is strengthening skills, connecting job-ready talent to industry and creating opportunities."
BMJ Publishing Group Ltd (London, United Kingdom)	thebmj (Career Focus) (see [26])	https://www.bmj.com/content/331/7516/s104.1.abstract (January 2020)	Blog	“*The BMJ* is defined by its mission: to work towards a healthier world for all. We share that global endeavour with millions of readers working in clinical practice, research, education, government, and with patients and the public too.”
California Institute of Behavioral Neurosciences and Psychology (CIBNP) (Fairfield, California)	Research Experience- California Institute of Behavioral Neurosciences and Psychology	https://www.cibnp.com/research-experience/ (2019)	Website	“Established in mid 2016, California Institute of Behavioral Neurosciences and Psychology has been helping understudies from everywhere throughout the world by means of Internet. CIBNP fills in as an online stage for understudies from various parts of the globe.”
Educational Commission for Foreign Medical Graduates (ECFMG) (Philadelphia, Pennsylvania)	Alternative Health Care Careers in Genetics	https://www.ecfmg.org/echo/webinars-june-2017.html (2020)	Website	“The ECFMG promotes quality health care for the public by certifying international medical graduates for entry into U.S. graduate medical education […].”
Educational Commission for Foreign Medical Graduates (ECFMG) (Philadelphia, Pennsylvania)	Alternative Careers in Health Care: Public Health	https://www.ecfmg.org/echo/alternative-careers-in-health-care-public-health.html (2020)	Website	As above
HospitalRecruiting.com (Bloomington, Indiana)	Career Options for International Medical Graduates [see [27]]	https://www.hospitalrecruiting.com/blog/2238/career-options-for-international-medical-graduates/ (January 2020)	Blog	HospitalRecruiting.com is a nationwide healthcare job board. Physicians, advanced practitioners, nurses, allied health professionals, and non-clinical healthcare professionals may use this website to browse open positions, apply to jobs, and to communicate with recruiters with active job openings
International Doctors Network (York, Ontario)	Alternative Careers in Health Promotion and Education for International Medical Graduates (IMGs) Fall 2019 Session	http://www.idnca.org/alternative-careers-in-health-promotion-and-education-for-international-medical-graduates-imgs-fall-2019-session/ (2019)	Website	The organization’s mission is to “facilitate the successful integration of Internationally Trained Doctors into the Canadian society as a whole.”
Katherine O’Brien Communications (N/R)	Katherine O’Brien Communications [see [28]]	https://katherineobrien.ca/writing-samples/back-up-plans-for-internationally-trained-doctors/ (N/R)	Blog	N/A
Metroland Media Group Ltd (Mississauga, Ontario)	Canadian Immigrant [see [29]]	https://canadianimmigrant.ca/careers-and-education/post-secondary-education/bridging-programs/program-empowers-internationally-trained-medical-doctors (N/R)	Blog	"[T]he magazine began with a mandate to "inform, educate and motivate" immigrants to Canada and assist them in their newfound journey. Since then, the magazine has grown to be the only national multi-platform brand for all immigrants to Canada, on topics from careers to education to settlement.”
North American College of Information Technology (NACIT) (Scarborough, Ontario)	North American College of Information Technology	http://www.nacollege.com/index.php/clinical-research/ (2018)	Website	NACIT’s “curriculum is based on intense job evaluation and input from potential employers. Through our skilled professional instructors and state-of-the-art technology, North American College produces graduates whose skills and knowledge have been recognized by employers, and who are well prepared to meet the challenges of today’s work environment.”
Ontario Council of Agencies Serving Immigrants (OCASI) (Ontario)	Settlement.org	https://settlement.org/alternative-jobs/doctor/ (2020)	Website	OCASI’s “mission is to achieve equality, access and full participation for immigrants and refugees in every aspect of Canadian life.?
Ryerson University (Toronto, Ontario)	The Chang School of Continuing Education	https://continuing.ryerson.ca/public/category/courseCategoryCertificateProfile.do?method=load&certificateId=207907 (N/R)	Website	Mission: “To be a leader in innovative, quality, lifelong learning that empowers adults to reach their life and career goals We are proud to be Canada’s largest, most successful continuing education program.”
USMLE (N/R)	USMLE Blog for Smart People	https://usmlesteps.blogspot.com/2008/10/alternate-pathways-for-foreign-medical.html (2008)	Blog	N/A
Vancouver Public Library (VPL) (Vancouver, British Columbia)	Skilled Immigrant InfoCentre	https://pwp.vpl.ca/siic/alternative-careers/physician-alternative-careers/ (January 2020)	Website	“The Skilled Immigrant InfoCentre is an online and in-person resource centre that helps newcomers to Canada find the information they need to get a job, explore careers or start a business. All of our services and resources are free and are created by staff at Vancouver Public Library.”

*N/A*  not applicable

Most of the web pages (*n* = 7) operated from Ontario, Canada. Two other Canadian web pages were from Alberta and British Columbia. Four web pages were operated from the USA, two from Pennsylvania (from the same organization) and one each from California and Indiana. Only one website was run from London, United Kingdom. Two websites did not report any location.

### Resources regarding alternative careers for IMGs

We also identified several resource-related websites for alternative careers for IMGs (Table 2). Most of them provided only information regarding the labour market outlook, wage information, potential employers, and recruitment services. Only one bridging programme was identified and described by two websites that help obtain careers in health research and management. The organizations listed a range of support services and activities on their websites, including language and communication enhancement training, skill development workshops, placement for gaining experience or hands-on training, and settlement services (Table 2).

**Table 2:** Alternative career pathways according to organization website

Organization	Type of alternative career pathway	Service/support provided
Agincourt Community Services Association (ACSA)	Health educator	Enhanced language training which focuses on:
	Health policy analyst	–Job search (researching job opportunities, resumes, interviews)
	Medical laboratory technician	–Workplace culture
	Public health inspector	–Healthcare ethics
	Clinical counselling	–Trends in the healthcare sector;
	Case management	–Unpaid work placements
	Individual and group therapy	–Occupation-specific language training (OSLT) programmes
	Group facilitation	–Employment guidance and search assistance programmes
	Social service for geriatrics	
	Health informatics (applying healthcare data to improve patient outcomes)	
	Dental hygiene	
	Sleep technology	
	Personal support worker	
	Medical laboratory technology	
	Medical radiation technology	
	Nutritionist	
	Pharmaceutical or medical supply sales	
	Clinical administration	
	Behavioral health coach	
	Holistic nutritionist	
	Chiropractor	
	Life enhancement assistant	
	Community health worker	
	Life enrichment coordinator	
	Corporate wellness professional	
	Massage therapist	
	Fitness instructor	
	Mental health coach	
	Fitness, recreation and wellness coach	
	Nutrition and wellness advisor	
	Health and wellness coach	
	Occupational health therapist	
	Health transitions coach	
	Physical therapist	
	Health and productivity analyst	
	Public health educator	
	Health improvement manager	
	Recreational therapist	
	Health management consultant	
	Vitamin and health food consultant	
	Health promoter	
	Weight loss consultant	
	Health service manager	
	Wellness and exercise instructor	
	Health, safety and wellness coordinator	
	Wellness coordinator	
	Healthy lifestyle specialist	
Alberta International Medical Graduate Association (AIMGA)	Mental health and counselling	Employment assessment
	Community health	Employment workshops/mentoring circles
	Emergency services	Individual career counselling session
	Management and health policy	Work/mentorship placement
	Research	Online portal to support
	Teaching	Research/journal club
	Healthcare related industries	
	Indigenous health	
	Technical skills	
BioTalent Canada	Clinical research associate	Relevant human resource service
	Clinical research data manager	
	Pharmacologist	
	Pre-clinical/clinical or field trial project manager	
	Project manager	
	Research assistant	
	Research director	
	Research manager	
	Research scientist	
BMJ Publishing Group Ltd. (see Pool 2005 [26])	Pharmaceutical medicine	N/R
	Analysis of healthcare stocks	
	Healthcare management	
California Institute of Behavioral	Research	N/R
Neurosciences and Psychology (CIBNP)		
Educational Commission for Foreign Medical Graduates (ECFMG)	Public health	Relevant information
	Genetics	
HospitalRecruiting.com (see [27])	Physician assistant	N/R
	Occupational therapist	
	Speech language pathologist	
	Pharmacovigilance and drug safety	
	Clinical research	
International Doctors Network (IDN)	Health promotion and education	N/R
Katherine O’Brien Communications	Medical radiation technologist	N/R
	Registered nurse	
	Medical assistant	
	Clinical research assistant	
	Medical screener	
	Clinical research associate	
	Clinical research coordinator	
	Health promoter/educator	
	Medical interpreter	
	Medical administrative assistant	
	Sleep technicians	
	Pathology assistants	
	Health policy analyst	
	Medical lab technician	
	Nurse aide	
	Blood donor clinic assistant	
	Personal support worker	
	Claims examiner/analyst/specialist	
	Medical underwriting	
	Mobile field examiner	
Metroland Media Group Ltd. (Canadian Immigrant) (see [29])	Medical research	Information on a bridging programme
	Health care management	
North American College of Information Technology (NACIT)	Clinical research	Relevant education programme
Ontario Council of Agencies Serving Immigrants (OCASI) [30]	Public health inspector	Immigrants and refugee settlement services
	Personal support worker	
	Medical laboratory technician	
	Health policy analyst	
	Health educator	
Ryerson University	Health research	Relevant education programme
	Health informatics	Bridging programme
	Data analysis	
	Health management	
USMLE [31]	Optometry	N/R
	Master’s in Public Health	
	Surgical assistants	
	Master/PhD degree in any basic medical sciences like pathology, pharmacology	
	Perfusion technology	
	Physician assistant	
Vancouver Public Library (VPL) [Skilled Immigrant InfoCenter]	Health care administrator	Online and in-person resource centre that offers employment guide which include:
	Health policy researchers, analysts	Labour market outlook
	Inspectors in public and environmental health and occupational health and safety	Wages/salary information
	Health care assistant (HCA) OR health care aides	Industry associations
	Medical laboratory assistant and medical laboratory technologists	Employment outlook
	Medical research	Credential evaluation links
	Occupation medicine	Industry journals
	Medical and health care product sales	– Industry websites
	Medical managers or administrators	
	Medical advisors or counselling	
	Medical communications	
	Health and /or mental health patient advocate	
	Government Department of Health (example: health planner or policy analyst)	

*N/R*  not reported

### Types of alternative careers extracted from the websites

**Fig. 2:**
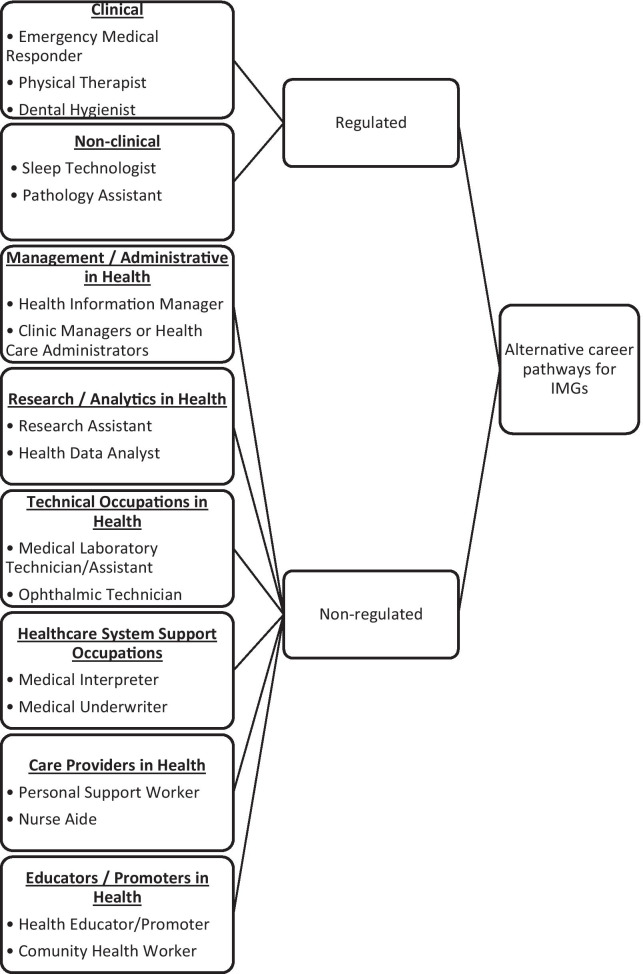
Taxonomy of alternative careers

We have extracted all the potential alternative careers for IMGs, for which information or other resources were available at the websites identified in this study (Table 2). We identified that some of these jobs require licenses from a regulatory body and some do not. Despite being non-physician professions, some jobs still require direct contact with the patient and help with diagnosis, treatment, and care of patients (i.e. clinical jobs). Other non-regulated jobs are categorized based on the major transferable skills they utilize. A categorization taxonomy of ACP jobs for IMGs in the health and wellness sector is illustrated in Fig. 2.

#### Regulated alternative careers

Depending on whether the regulated jobs require direct contact and care with patients, we further classified them into two types: (1) clinical and (2) non-clinical. The clinical jobs were those related to providing bedside treatment to patients. Many alternative careers fall into this category, such as a nurse, chiropractor, and respiratory therapist.

Non-clinical jobs are associated with the diagnosis and management of patients, but are not associated with any bedside treatment procedures and responsible for making any treatment-related decisions for patients. These jobs may occur in clinical or laboratory settings. Examples include medical laboratory technologist, pathology assistant, and speech-language pathologist.

#### Non-regulated alternative careers

Most alternative career choices do not require licensure or certification from a regulatory body. However, as they are quite diverse we have attempted to classify them into certain categories. These include (1) management and administrative jobs that require planning and coordination of various healthcare services and health centres (e.g., health programme manager); (2) research and analysis jobs that require analysis of health-related data and research (e.g., clinical research associate); (3) technical occupations in health that require using some technical skills for healthcare services (e.g., ophthalmic technician); (4) healthcare system support occupations that provide support to the healthcare system (e.g., medical interpreter); (5) care providers in health who provides personal care and companionship for seniors, and persons with disabilities (e.g., rehabilitation aide), and (6) educators/promoters in health who promotes community health and wellness (e.g., community health worker).

### Identification of transferable of IMGs

The websites have also pointed out the transferable skills of IMGs that can be utilized in the above-mentioned alternative jobs. Transferable skills for IMGs are those skills that IMGs generally obtain during their medical education, training, and experience while working as physicians, which, in turn, can also be adapted for careers in alternative professions. A range of transferable skills was identified on the websites we reviewed and listed in Table 3. Some are related to clinical knowledge and skills, such as medical terminology [28, 32, 33], accountability/professionalism [7, 8], flexibility [7, 8], and patient care [7, 8, 28]. Some essential skills were mentioned as transferable skills of IMGs such as compassion and understanding [7, 8, 33], excellent communication skills [7, 8, 27–29, 32–35], and teamwork [7, 8] can be transferred to healthcare supporting jobs. The websites also reported that through their experience as a physician IMGs also gained some administrative skills [7, 8, 28, 32, 33], planning and leadership proficiencies, decision-making capability [33], and advocacy skills [7, 8]. Skills such as interpersonal and analytical skills [7, 8], attention to details [7, 8], problem-solving skills [33], critical thinking [7, 8], and research and report writing [7, 8, 24, 27–29, 33–38] are transferable to research and analytical jobs. Background knowledge and skills of IMGs, namely, science and specific medical knowledge [7, 8, 27, 28, 34, 37, 39] and technical and surgical skills [7, 8, 28], can be employed in technical occupations that are potential alternative careers for IMGs. Moreover, many IMGs also have experience in education/teaching [7, 8, 32, 34, 35, 37], often as teachers in medical schools and patient educators.

**Table 3:** Transferable skills of IMGs

Organization	Transferable skills
Agincourt Community Services Association (ACSA)	Science and specific medical knowledge
BioTalent Canada	Research and report writing
Alberta International Medical Graduate Association (AIMGA)	Administrative
	Research and report writing
	Compassion and understanding
	Critical thinking
	Management
	Detail oriented accountability/professionalism
	Flexibility
	Excellent communication skills
	Patient care
	Interpersonal and analytical skills
	Advocacy skills
	Teamwork
	Science and specific medical knowledge
	Education/teaching
	Technical and surgical skills
Vancouver Public Library (VPL) [Skilled Immigrant InfoCenter]	Administrative
	Medical terminology
	Research and report writing
	Compassion and understanding
	Strong planning and leadership skills
	Excellent communication skills
	Decision-making
	Problem solving skills
International Doctors Network (IDN)	Administrative medical terminology
	Excellent communication skills
	Education/Teaching
California Institute of Behavioral	Research and report writing
Neurosciences & Psychology (CIBNP)	
Educational Commission for Foreign Medical Graduates (ECFMG)	Research and report writing
	Excellent communication skills
	Education/teaching
	Science and specific medical knowledge
North American College of Information Technology (NACIT)	Research and report writing
BMJ Publishing Group Ltd. (see Pool [26])	Management
Metroland Media Group Ltd. (Canadian Immigrant) [see [29]]	Research and report writing
	Excellent communication skills
HospitalRecruiting.com (see [27])	Research and report writing
	Excellent communication skills
	Science and specific medical knowledge
Katherine O’Brien Communications	Administrative
	Medical terminology
	Research and report writing
	Excellent communication skills
	Patient care
	Science and specific medical knowledge
	Technical and surgical skills

## Discussion

Through our I-scan we identified the websites of the information and capacity-building resources and extracted the supports available for the IMGs’ pursuit of alternative careers. We found that most of the supports are non-specific to IMGs and rather focused on general employment-related supports such as language training, job search, and Canadian work environment-related information and training. We have only found one bridging programme that specifically supports and provides training to IMGs for building their capacity for certain alternative careers. However, many possible alternative jobs were mentioned and information regarding how to avail those careers was described to some extent. Therefore, we attempted to extract the alternative careers and the transferable skills and put them into certain categories that will help cluster the jobs requiring similar transferable skills and help IMGs and their stakeholders take action to their capacity accordingly. A range of transferable skills including essential skills such as administrative and leadership, and specific health-related technical knowledge and expertise such as knowledge of medical terminology was mentioned in the webpages. Identifying and nurturing those can help target relevant jobs and employ these valuable skills and improve professional integration.

Our search did not reveal extensive resources and initiatives to bolster IMGs’ pursuit of job market integration through alternative paths outside of the physician profession. Possible explanations for the limited information could be the concept of employing unlicensed IMGs into health and wellness through alternative careers is relatively novel. Moreover, IMGs come to this country with the dream of becoming a practising physician and spent most of their time and energy in the pursuit of physician licensure. Unfortunately, when many of them fail in the pursuit of their long-cherished dream after years of endeavour, only then they think about alternative career. But at that moment they do not have enough time and energy, and often burdened with financial and family responsibilities that hinder them to give more efforts for decent alternative career pursuit [7, 8, 40, 41]. We did not put any country or region restriction in our search, however, we noticed that available information sources are geographically condensed in Canada followed by the United States. This probably reflects the fact that despite being popular destinations for IMGs, the success rate for integrating as a physician Canada (15–30%) is much lower than the other common IMG destinations in the world such as the USA, the UK and Australia (50–80%) [34, 37, 40–44] The Canada-centricity of the findings of our search indicates that Canada is the most unfavourable host country for integrating IMGs as a physician and requires systematic and strong support the most for the IMGs in their alternative career pursuit.

There is a lack of comprehensive information about the processes IMGs go through when searching for work and the factors that influence their career decisions. Exploring the hiring process from the perspective of employers would deepen our understanding of their decisions with regard to hiring IMGs [45]. Additional research is also needed to develop evidence-based strategies to support IMGs with their career choice process [7, 8]. A decision support tool might prove helpful for IMGs in their efforts to find and pursue an alternative career where they can use their existing skills [40, 41]. We also could not find any indication that cultural backgrounds, any suitable previous training for alternative careers, socioeconomic constraints in the new country, or other factors have been considered when working on ACPs for IMGs. Furthermore, it seems the IMG population has been considered a single homogeneous population, but in reality, it is very heterogeneous [46]. The heterogeneity factor needs to be incorporated in our understanding in this area.

Despite being a non-conventional approach to knowledge synthesis this I-scan study has several strengths. To extract the non-academic information, which follows no structured organization of knowledge repositories and databases, we have used a methodologically sound and transparent process to identify and map the information. We have gathered a team of researchers with expertise in the study area and knowledge syntheses methodologies who designed and conducted the study. We have also engaged stakeholders and citizen researchers’ from IMGs and IMG serving organizations to validate and contextualize the data, and their contributions throughout the project made the findings applicable and highly relevant to stakeholders and knowledge users.

Notwithstanding the strengths, some limitations need to be recognized. Given the complexity and breadth of definitions for ACPs, our search terms may have resulted in missing certain web pages. Also, despite using the incognito mode of the browsers to carefully avoid the reviewers’ location and personal web activity history influencing the search results, it cannot be guaranteed that the search results were not absolutely influenced by the IP (Internet Protocol) address of the search. This could have skewed the origin country of the majority of the identified websites in this study to Canada. However, we argue that our flexible approach to search terms ensured a reasonable breadth of relevant web page identification. Another limitation is that our search was restricted to the English language. Thus, our findings may not be generalizable to non-English speaking countries, e.g., in Germany, where more and more IMGs immigrate to fill the need for physicians, and in 2016 IMGs constituted 11% of the German medical workforce, with the majority coming from Southeastern Europe and Syria [47].

## Conclusion

In conclusion, this study serves as an initial step towards establishing a practical knowledge base for identifying research and development gap including strategic support and programmes for facilitating IMGs’ pursuit of alternative careers. Our study indicates that there is only a handful of information resources and support initiatives available for IMGs’ pursuit of alternative career. Moreover, despite IMGs more or less struggle to pursue their primary career choice, i.e. becoming a licensed physician in many countries, the information and support regarding that were more limited to Canada. This could be made more common to the USA, the UK, and other countries. Therefore, more systemic policies and IMG specific and ACP-focused employment support programmes are needed to be built. Our findings can help IMGs, stakeholders, and policy decision-makers make more informed personal and policy decisions.

### Policy recommendations

We recommend to increase research focus towards understanding the decision-making aspects for IMGs regarding career choices towards integration in host country job market. In addition, the benefit and impact of ACPs need to be shared with IMGs and their potential employers through meaningful engagement. Additionally, more systematic research is needed to understand the transferable skills that the IMGs possess and how those can be cultivated to make them more applicable in the ACPs.

## Data Availability

The manuscript contains all data.
